# Age at Menarche and Risk of Hypertensive Disorders of Pregnancy: A Retrospective Cohort Study

**DOI:** 10.3390/clinpract16020032

**Published:** 2026-01-29

**Authors:** Erick Ordoñez-Villordo, Monica Alethia Cureño-Díaz, Erika Gómez-Zamora, Miguel Trujillo-Martínez, Ricardo Castrejón-Salgado, Fani Villa-Rivas, Rocío Castillo-Díaz, Nadia Velázquez-Hernández, Juan Carlos Fernando Sánchez-Velázquez, Ximena Solis-Gómez, José Ángel Hernández-Mariano

**Affiliations:** 1 Faculty of Medicine, Puebla State University of Health, Puebla of Zaragoza 72000, Mexico; 2 Department of Institutional Intelligence in Oncological Health, National Institute of Cancerology, Mexico City 14080, Mexico; 3 Department of Medical Management, Hospital Juarez of Mexico, Mexico City 07760, Mexico; 4 General Hospital with Family Medicine Unit Number 7, Mexican Social Security Institute, Cuautla 62740, Mexico; 5 Health Research Coordination, Mexican Social Security Institute, Cuernavaca 62000, Mexico; 6 Faculty of Nursing and Midwifery, Juarez University of the State of Durango, Durango 34217, Mexico; 7 Scientific Research Institute, Juarez University of the State of Durango, Durango 34000, Mexico; 8 Nursing School, Hospital Juárez de México, Mexico City 06090, Mexico; 9 School of Medicine, National Autonomous University of Mexico, Mexico City 04360, Mexico; 10 Department of Research, Hospital Juarez of Mexico, Mexico City 07760, Mexico

**Keywords:** menarche, hypertensive disorders pregnancy, pregnancy, body mass index

## Abstract

**Background/Objectives**: Hypertensive disorders of pregnancy (HDP) remain a major contributor to maternal morbidity and mortality worldwide, yet early-life reproductive factors such as age at menarche have been insufficiently explored in relation to HDP. Therefore, we aimed to evaluate the association between age at menarche and the risk of HDP in a cohort of Mexican pregnant women. **Methods**: We conducted a retrospective cohort study among 1344 women with singleton pregnancies receiving care at a tertiary hospital in Mexico City in 2024. Age at menarche was categorized as <12, 12–14, and >14 years. HDP diagnoses were extracted from clinical records. Poisson regression with robust variance was used to estimate adjusted risk ratios (RRs). Sensitivity analyses included alternative menarche categorizations and restricted cubic spline models. Counterfactual mediation analyses assessed indirect effects through reconstructed prepregnancy BMI and gestational diabetes. **Results**: Both early (<12 years) and late (>14 years) menarche were associated with higher HDP risk than the 12–14-year reference (adjusted RR = 1.81; 95% CI 1.42–2.30, and 1.74; 95% CI 1.27–2.38, respectively). Spline models confirmed a U-shaped association. Mediation analyses indicated that prepregnancy BMI did not meaningfully mediate the association for either early or late menarche (<5% mediated). Gestational diabetes explained a modest proportion of the association for early menarche (≈14%), but not for late menarche. **Conclusions**: Age at menarche showed a robust U-shaped association with HDP, mostly independent of adiposity and gestational diabetes, within the limits of the available measurements. Incorporating pubertal timing into routine reproductive history taking may enhance contextual risk assessment for HDP.

## 1. Introduction

Hypertensive disorders of pregnancy (HDP) comprise a spectrum of conditions that manifest as elevated blood pressure during pregnancy and represent a leading cause of maternal and perinatal morbidity and mortality worldwide [1]. In addition to their immediate impact, HDP increases the risk of serious complications such as intrauterine growth restriction, preterm birth, HELLP syndrome, placental abruption, and maternal or neonatal death [2,3]. Women who experience these disorders are also more likely to develop chronic hypertension, cardiovascular disease, and metabolic syndrome later in life [4]. Clinically, these entities are primarily distinguished by their presentation: gestational hypertension is characterized by elevated blood pressure after 20 weeks of gestation in the absence of proteinuria; preeclampsia includes proteinuria or evidence of organ dysfunction; eclampsia adds the presence of seizures; superimposed preeclampsia occurs when a woman with pre-existing hypertension develops new signs of renal, hepatic, or hematologic involvement during pregnancy. Although their manifestations vary, they all share pathophysiological mechanisms related to endothelial dysfunction, systemic inflammation, and perfusion abnormalities.

The prevalence of pregnancy complications varies across populations, healthcare systems, and diagnostic criteria. Recent estimates indicate that the global number of prevalent cases increased from approximately 6.15 million in 1990 to 36.10 million in 2021, affecting an estimated 10–22% of pregnancies worldwide [5].

Despite this substantial rise in absolute numbers, age-standardized prevalence rates have remained relatively stable over time. This pattern reflects strong demographic pressures, particularly population growth, combined with persistent inequalities in maternal health [5]. Notably, low- and middle-Socio-demographic Index (SDI) regions (a composite indicator of income, education, and fertility) bear a disproportionate share of the burden, showing higher prevalence and slower declines than high-SDI settings [6]. These disparities underscore the vulnerability of countries in Latin America and other resource-limited contexts.

In Mexico, national hospital discharge data also document a significant burden of these disorders. A recent longitudinal analysis of routine information systems showed that rates increased from 0.54 to 2.42 cases per 100 live births between 2000 and 2020 among women without social security [7]. Additionally, facility-based studies from different Mexican states report prevalences of hypertensive disease of pregnancy ranging from approximately 5% to 10% of deliveries, which aligns with estimates from other middle-income settings [8,9].

While factors such as advanced maternal age, obesity, a history of hypertension, and pre-existing metabolic conditions are well-established determinants of HDP [10] growing attention has been directed toward early-life reproductive factors, particularly the timing of menarche. Menarche is typically classified as early if it occurs before age 12 and as late if it occurs after age 15. From a physiological perspective, precocious puberty involves prolonged exposure to estrogens, progesterone, and androgens, which promote visceral fat accumulation, endothelial dysfunction, and increased sympathetic activity [3,11]; these mechanisms are also implicated in the pathogenesis of HDP [12].

Even with biologically plausible pathways suggesting an association between pubertal timing and cardiovascular regulation, epidemiological evidence linking age at menarche to HDP remains limited and inconsistent. Several studies have reported that women with early menarche are more likely to develop hypertension during both premenopausal and postmenopausal stages [13,14,15], and some evidence suggests that obesity may partially mediate this relationship [14,15,16]. Conversely, late menarche has also been linked to adverse cardiovascular profiles. One investigation found that women who experienced menarche after age 14 had a higher risk of hypertension [17], while another reported that those with menarche at or after age 16 exhibited significantly higher systolic and diastolic blood pressure levels.

In line with these findings, emerging evidence supports a nonlinear, U-shaped association, in which both early (≤12 years) and late (≥16 years) menarche are associated with an increased risk of hypertension in adulthood, with age 14 as the reference category [18]. This pattern suggests that deviations at either end of the menarcheal age distribution may reflect distinct biological or environmental processes influencing long-term cardiovascular regulation. However, not all studies have confirmed these patterns. At least one investigation reported no significant association between age at menarche and adult blood pressure [19], suggesting that the influence of pubertal timing on cardiovascular health may vary across populations and study methodologies.

On the other hand, evidence linking age at menarche to HDP is even more limited and remains inconclusive. Recent evidence suggests that early menarche may be an essential marker of susceptibility to hypertensive conditions in pregnancy. A large prospective cohort from China reported that women with menarche at ≤13 years had a significantly higher risk of both gestational hypertension and preeclampsia compared with those with later menarche [20]. Similarly, a UK study found that earlier menarche was associated with higher mean arterial pressure during pregnancy, an association partly explained by pre-pregnancy adiposity [21]. In contrast, a case–control study from Ethiopia found no significant relationship between menarcheal age and HDP [22].

In Latin America, the association between age at menarche and hypertensive complications of pregnancy has been scarcely explored. To date, no studies in Mexico have specifically examined this relationship, despite the documented generational decline in menarcheal age and the continued high prevalence of gestational hypertension and preeclampsia in the region. Clarifying whether pubertal timing serves as an early reproductive indicator of susceptibility to these conditions could facilitate the identification of women who may benefit from more focused screening, preconception counseling, and preventive strategies.

Importantly, age at menarche is not conceptualized here as an isolated causal determinant, but rather as a stable reproductive marker that integrates genetic, nutritional, metabolic, and psychosocial influences accumulated across childhood and adolescence. From a life-course perspective, pubertal timing may capture underlying developmental trajectories that later shape cardiovascular vulnerability, including adaptive responses during pregnancy. Evaluating this marker within specific epidemiological contexts, such as Mexico, where early-life metabolic risk and HDP are both prevalent, may therefore provide insight into population-level patterns of risk.

Despite this life-course conceptualization, most existing studies on the association between age at menarche and hypertensive outcomes come from large prospective cohorts, typically using validated self-reported menarcheal age and objectively measured pre-pregnancy body mass index. By contrast, data from routine clinical settings (particularly in middle-income countries) remain scarce. In these contexts, reproductive history and anthropometric information are often obtained retrospectively from medical records, reflecting everyday obstetric practice rather than research-optimized conditions. Assessing whether the association between pubertal timing and HDP can be observed under such conditions is essential for evaluating its relevance in real-world clinical care. Hence, we aimed to examine the relationship between age at menarche and hypertensive outcomes during pregnancy among Mexican women.

## 2. Materials and Methods

### 2.1. Design and Study Population

This study is a secondary analysis of a retrospective database generated as part of a larger project approved by the Research and Ethics Committee of the Hospital Juárez de México (approval number 078-24-I). The parent project authorized the review of obstetric and perinatal medical records to evaluate maternal and neonatal outcomes. We conducted a retrospective cohort study based on medical records of pregnant women who attended a tertiary care hospital in Mexico City between 1 January and 31 December 2024. The institution provides specialized care to individuals without social security coverage.

Eligible participants were women aged 18–45 years with singleton pregnancies and complete information on age at menarche and HDP. We excluded women with pregestational hypertension, chronic kidney disease, multiple gestations, or incomplete data on exposure or outcomes. HDP, comprising gestational hypertension, preeclampsia, and eclampsia, was obtained directly from the diagnoses recorded by attending physicians as part of routine clinical care, without reclassification by the research team.

Of the 1439 eligible records identified, 1344 were included in the analysis, corresponding to an inclusion rate of 93.4% after applying the exclusion criteria (Figure S1).

### 2.2. Data Collection and Study Variables

Data extraction was conducted through a systematic review of paper-based clinical files, as no electronic medical record system was available. Authorization from the Clinical Records Department permitted access to all eligible charts. To ensure reliability in case selection and variable abstraction, an independent review of 15% of records (n = 135) was performed before applying exclusion criteria. Interobserver agreement, assessed using Cohen’s kappa coefficient, was 0.85, indicating substantial concordance among reviewers.

HDP, comprising gestational hypertension, preeclampsia, and eclampsia, was identified directly from diagnoses recorded in the medical records as part of routine clinical care. Diagnoses were made by attending obstetricians and gynecologists in accordance with the American College of Obstetricians and Gynecologists (ACOG) criteria in effect during 2024. The research team did not reclassify outcomes. Although clinical practice generally follows standardized ACOG definitions, some degree of inter-clinician variability in diagnostic interpretation cannot be completely excluded.

Age at menarche was obtained from routinely collected obstetric and gynecologic records. During the initial prenatal assessment, women were asked to report their age at first menstruation, which was recorded in years in the clinical history by trained healthcare personnel. For the purposes of this study, age at menarche was analyzed both as a continuous variable and as predefined categories (<12, 12–14, and >14 years). Records with missing age-at-menarche information were excluded from the analytical sample. No implausible values were identified within the observed range.

The study collected information on key covariates documented in the medical records. Sociodemographic variables included maternal age, marital status, educational level, and household income. Obstetric history was abstracted from clinical files and included parity and any documented history of hypertensive disorders in prior pregnancies. Clinical and familial information encompassed the pre-pregnancy BMI category and a family history of hypertension. The presence of gestational diabetes in the current pregnancy was also recorded as part of routine obstetric care. Given that anthropometric measurements from early pregnancy were not consistently available, we reconstructed baseline BMI using self-reported weight before conception and the documented height in the clinical file. This method is commonly used in retrospective obstetric studies when complete prenatal measurements are unavailable.

### 2.3. Statistical Analysis

We described participants’ characteristics using standard descriptive statistics. Categorical variables were summarized as frequencies and percentages, while continuous variables were presented as medians with interquartile ranges (IQRs) because they did not follow a normal distribution according to the Shapiro–Wilk test. Comparisons between women with and without HDP were performed using Pearson’s chi-square test for categorical variables and the Mann–Whitney U test for continuous variables.

To estimate the association between age at menarche and HDP, we calculated risk ratios (RRs) and 95% confidence intervals (CIs) using Poisson regression models with robust variance. Age at menarche was categorized into three groups (<12, 12–14 [reference], and >14 years).

In our sensitivity analyses, we examined alternative categorizations of age at menarche based on definitions commonly used in epidemiological and cardiometabolic research. Several large cohort studies and meta-analyses have classified very early menarche as <11 years due to its established association with adverse metabolic outcomes [23,24]. In contrast, menarche at 12–13 years is widely considered the modal or average timing in population-based studies and is frequently used as the reference category [25,26,27]. Accordingly, we re-estimated the association using two established specifications: <11 /11–14/ >14 years, and ≤11 /12–13/ ≥14 years. These sensitivity models help assess whether the observed association is robust to different, literature-based definitions of pubertal timing.

Additionally, we modeled age at menarche as a continuous exposure using restricted cubic splines (RCS) to evaluate potential non-linear associations with HDP. The spline model specified four knots located at the 5th, 35th, 65th, and 95th percentiles of the menarcheal age distribution, corresponding to approximately 10, 11, 13, and 15 years, respectively, using 12 years as the reference value. This knot placement followed commonly used recommendations for spline modeling in epidemiologic studies, balancing flexibility with model stability and avoiding overfitting at the extremes of the distribution (Table S1). Departure from linearity was assessed using Wald tests for the joint significance of the non-linear spline terms.

To assess the robustness of the association across clinically relevant subgroups, we conducted stratified analyses by parity (nulliparous vs. multiparous) and estimated stratum-specific risk ratios using the same adjusted Poisson regression models. In addition, potential effect modification by pre-pregnancy BMI (<25 vs. ≥25 kg/m^2^) was evaluated by including interaction terms between age at menarche and BMI category in the adjusted models. Stratum-specific estimates were derived from these models to aid interpretation.

To explore potential pathways linking age at menarche to HDP, we conducted counterfactual-based mediation analyses. Two clinically relevant metabolic factors were evaluated as potential mediators: prepregnancy body mass index (categorized as <25 vs. ≥25 kg/m^2^) and gestational diabetes. Given the requirements of the counterfactual framework for binary exposures, separate mediation models were fitted comparing early menarche (<12 years) and late menarche (≥15 years) with the reference category (12–14 years). Because of the retrospective design, reconstructed measurement of prepregnancy BMI, and the assumption rather than empirical verification of temporal ordering, mediation analyses involving both prepregnancy BMI and gestational diabetes were conducted for exploratory purposes and should not be interpreted as evidence of causal mediation.

Mediation analyses were conducted using Stata version 19.5 and the “mediate” command, which implements a counterfactual-based framework to decompose the total effect of an exposure into direct and indirect components, as commonly applied in epidemiologic mediation research [28,29]. In line with the main analyses, results are presented as risk ratios (RRs) using the “estat irr” postestimation command, along with the proportion of the total effect attributable to the indirect pathway. Given the retrospective design and the exploratory nature of the mediation analyses, we assessed the robustness of the estimates using nonparametric bootstrap resampling with 1000 replications and a fixed random seed to ensure reproducibility. Bootstrap-based confidence intervals were consistent with those obtained from the primary models, supporting the stability of the estimated effects. Accordingly, the mediation results from the original models are reported.

All models were adjusted for confounding factors. The selection of confounders was guided by directed acyclic graphs (DAGs) [30,31]. The minimum adjustment set included maternal age, educational level, monthly family income, and a family history of hypertension. (Figure S2). To evaluate the potential influence of unmeasured confounding, we estimated E-values for the observed associations between age at menarche and HDP. The E-value quantifies the minimum strength of association that an unmeasured confounder would need to show with both the exposure and the outcome, after accounting for the measured covariates, to fully explain the observed effect estimates [32].

A *p*-value of <0.05 was used as the criterion for statistical significance in all regression models and hypothesis tests. All analyses were conducted using Stata statistical software, version 19.5 (StataCorp, College Station, TX, USA).

## 3. Results

Table 1 describes the baseline profile of the study cohort. The median maternal age was 25 years (interquartile range: 10 years). Just over half of the participants had completed lower secondary education (51.8%), and most were married or living with a partner (97.2%). The median monthly household income was 512 units (IQR: 73.6). Almost half of the women (47.0%) had one or two previous births, whereas 42.5% were nulliparous.

Regarding clinical history, 10.3% of participants had gestational diabetes in the current pregnancy, 23.7% reported a family history of hypertension, and 26.6% a family history of diabetes. Alcohol and tobacco consumption were uncommon (5.5% and 10.5%, respectively). Prepregnancy overweight or obesity (BMI ≥ 25 kg/m^2^) was observed in 41.7% of the sample. Age at menarche showed substantial variability: 61.1% experienced menarche before age 12, 26.9% between ages 12 and 15, and 12.0% after age 15.

Among the 254 women who developed HDP, 172 had gestational hypertension (67.7%), 78 had preeclampsia (30.7%), and four experienced eclampsia (1.6%). Women with HDP differed from those without these conditions in several key characteristics. They tended to be slightly older and had a substantially higher frequency of previous HDP. Gestational diabetes was also more common among women with HDP. In addition, early menarche was more common among cases than among controls. By contrast, no meaningful differences were observed between groups in education level, marital status, household income, parity, lifestyle behaviors, or family history of diabetes or hypertension.

In the unadjusted analysis, both early and late menarche were associated with an increased risk of HDP. Women with menarche before age 12 had 1.80 times the risk of HDP (95% CI 1.41–2.29), and those with menarche after age 14 had 1.75 times the risk (95% CI 1.28–2.39) compared with the reference group (12–14 years). The crude prevalence of HDP was 14.4% among women with menarche at 12–14 years, compared with 26.0% among those with early menarche (<12 years) and 25.3% among those with late menarche (>14 years), providing absolute context for these relative estimates.

After adjusting for confounding variables, the associations remained significant and essentially unchanged (early menarche: adjusted RR = 1.81; 95% CI 1.43–2.30; late menarche: adjusted RR = 1.74; 95% CI 1.27–2.37) (Table 2). For early menarche, the E-value for the adjusted point estimate was 3.03, and 2.21 for the lower confidence limit, indicating that an unmeasured confounder would need to be associated with both the exposure and HDP by risk ratios of at least this magnitude to fully explain the observed association. For late menarche, the corresponding E-values were 2.73 for the point estimate and 1.70 for the lower confidence limit, suggesting that residual confounding would need to be moderately strong to account for the effect.

To assess the robustness of the categorical findings, we conducted sensitivity analyses using alternative definitions of age at menarche. When using 11–14 years as the reference, both very early (<11 years) and late (>14 years) menarche were associated with higher HDP risk (RR = 1.49 and 1.50, respectively). A second specification using ≤11, 12–13 (reference), and ≥14 years showed similar associations (RR = 1.79 and 1.50, respectively). These results support the robustness of the U-shaped relationship observed in the primary analysis (Tables S2 and S3).

To further assess the robustness of the association, we conducted stratified analyses by parity. The association between age at menarche and HDP was broadly consistent across nulliparous and multiparous women. Early menarche (<12 years) was associated with an increased risk of HDP in both parity strata, while late menarche (>14 years) showed a similar association, with wider confidence intervals among nulliparous women. Stratum-specific estimates are presented in Table S4.

We then evaluated whether the association between age at menarche and HDP differed according to pre-pregnancy BMI category. No statistically significant interaction was observed (global *p* for interaction = 0.63), indicating that the association did not vary materially across BMI categories. Stratum-specific estimates are shown in Table S5.

**Figure 1:**
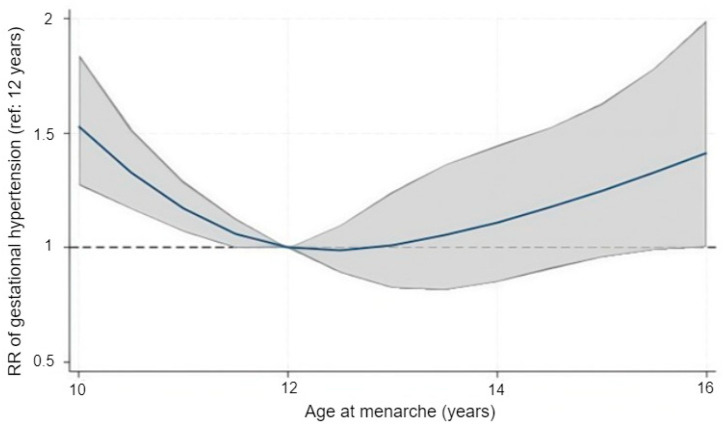
Adjusted association between age at menarche and the risk of gestational hypertension modeled with restricted cubic splines. The solid line represents the adjusted relative risk (RR) and the shaded area the 95% confidence interval, using menarche at 12 years as the reference value (RR = 1.0). The dashed horizontal line indicates the null value (RR = 1.0). Models were adjusted for maternal age, education, household income, and family history of hypertension.

After observing consistent results across the categorical models, we explored the functional shape of the association using restricted cubic splines. Treating age at menarche as a continuous variable revealed a distinctly U-shaped pattern in relation to HDP risk (Figure 1). Using 12 years as the reference, the adjusted relative risk remained close to 1.0 for menarche at 12–13 years but rose steadily at both younger and older ages. The predicted risk was higher around ages 10–11 and again from approximately 14 years onward. Confidence intervals were wider near the distributional extremes, reflecting the smaller number of observations in those ranges. The spline model reproduced this curvilinear trend and provided statistical support for non-linearity, with the spline terms jointly significant (Wald χ^2^(3) = 21.3, *p* = 0.0001).

To evaluate whether adiposity-related pathways contributed to the association between age at menarche and HDP, we performed exploratory mediation analyses using prepregnancy BMI as the proposed mediator.

**Figure 2:**
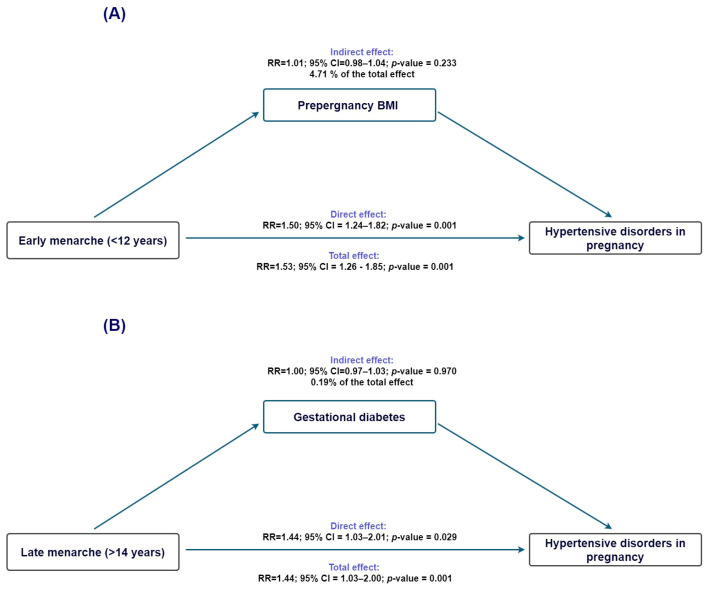
Counterfactual mediation analysis of the association between age at menarche and hypertensive disorders of pregnancy. (**A**) Estimated overall, direct, and indirect effects for early menarche (<12 years), using prepregnancy BMI as the proposed mediator. The overall effect was statistically significant, while the indirect pathway through BMI was minimal. (**B**) Corresponding estimates for late menarche (>14 years), for which neither the overall nor the indirect effects reached statistical significance. Ninety-five percent confidence intervals are displayed.

For early menarche (<12 years; Figure 2A), the total effect on HDP was statistically significant (TE IRR = 1.53, 95% CI 1.26–1.86), and the direct effect remained virtually unchanged after accounting for BMI (NDE IRR = 1.51, 95% CI 1.24–1.83). The indirect effect was small and nonsignificant (NIE IRR = 1.02, 95% CI 0.99–1.04), with only 4.7% of the association mediated (95% CI −3.1% to 12.5%), indicating that BMI did not meaningfully account for the observed association. For late menarche (>14 years; Figure 2B), the total effect was also significant (TE IRR = 1.45, 95% CI 1.04–2.01); however, the indirect effect through BMI was essentially null (NIE IRR = 1.00, 95% CI 0.97–1.03), and the proportion mediated was negligible (0.2%, 95% CI −9.8% to 10.2%). Thus, for both early and late menarche, the association with HDP appears to operate independently of BMI-related pathways.

**Figure 3:**
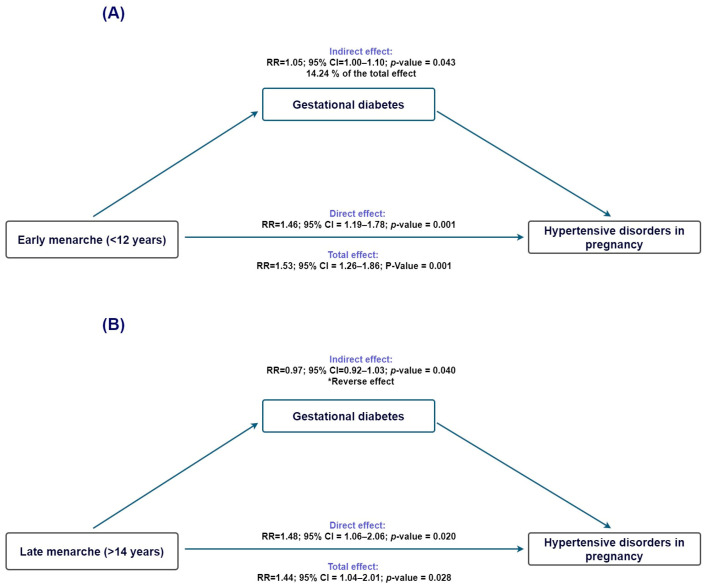
Mediation analysis evaluating gestational diabetes as a mediator in the association between age at menarche and hypertensive disorders of pregnancy. (**A**) Early menarche (<12 years): estimated direct and indirect effects through pregestational BMI. (**B**) Late menarche (>14 years): estimated direct and indirect effects through pregestational BMI. Ninety-five percent confidence intervals are displayed. * Indirect effect in the opposite direction of the total effect.

To investigate whether gestational diabetes might contribute to the pathway linking age at menarche with HDP, we conducted causal mediation analyses using gestational diabetes as the mediator. For early menarche (<12 years; Figure 3A), the total effect on HDP was statistically significant (TE risk ratio = 1.54; 95% CI 1.27–1.86), and the direct effect remained elevated after accounting for gestational diabetes (NDE risk ratio = 1.46; 95% CI 1.20–1.78). The indirect effect operating through gestational diabetes was small but statistically significant (NIE risk ratio = 1.05; 95% CI 1.00–1.11), corresponding to an estimated proportion mediated of 14.2% (95% CI −0.2% to 28.7%).

When examining late menarche (>14 years; Figure 3B), the total and direct effects on HDP were again statistically significant (TE risk ratio = 1.45; 95% CI 1.04–2.01; NDE risk ratio = 1.48; 95% CI 1.06–2.06). In contrast, the indirect effect through gestational diabetes was close to null (NIE risk ratio = 0.98; 95% CI 0.92–1.03), and the proportion mediated could not be reliably estimated because the direct and indirect effects operated in opposite directions, indicating no meaningful mediation through gestational diabetes.

## 4. Discussion

In this retrospective cohort, we observed a consistent U-shaped association between age at menarche and HDP, with higher risk at both early and late ages of onset. This pattern was robust across categorical, continuous, and spline-based models and persisted after adjustment for multiple sociodemographic and clinical factors. Mediation analyses did not support a meaningful indirect effect through prepregnancy body mass index or gestational diabetes. Notably, nearly one-third of participants reported menarche before age 12, consistent with national evidence of a secular decline in menarcheal age among Mexican women. Previous studies conducted in different regions of the country report mean ages at menarche ranging from 11.3 to 12.0 years, supporting the representativeness of our cohort and the broader demographic shift toward earlier reproductive maturation in Mexico [33,34,35].

From a life-course perspective, menarcheal timing itself is unlikely to be a direct biological cause of HDP. Instead, it represents a salient reproductive milestone that integrates genetic, nutritional, metabolic, and psychosocial influences accumulated across childhood and adolescence. Within this life-course framework, age at menarche can be interpreted as a proxy marker of earlier biological programming relevant to later cardiovascular adaptation during pregnancy [24,36,37].

Additional analyses support the robustness of the observed association. Stratified analyses by parity showed broadly consistent associations between age at menarche and HDP among nulliparous and multiparous women, suggesting that the observed relationship is not restricted to a specific obstetric subgroup. Likewise, formal assessment of multiplicative interaction with pre-pregnancy BMI did not indicate meaningful effect modification, with comparable risk estimates across BMI categories. This finding is consistent with epidemiological evidence indicating that associations between age at menarche and adverse cardiovascular outcomes often persist after accounting for adiposity, suggesting that factors beyond adiposity alone may contribute to these relationships (i.e., early life programming or long-term endocrine or metabolic regulation) [38]. Taken together, these results suggest that age at menarche may reflect broader developmental characteristics associated with HDP risk, rather than acting solely through parity- or adiposity-related pathways.

Clinically, these relative associations translate into meaningful differences in risk. The observed risk of HDP among women with menarche at 12–14 years was 14.49%, whereas applying the adjusted risk ratios yields an illustrative risk of approximately 25–27% among women with early or late menarche, corresponding to an absolute risk difference of roughly 10–12 percentage points.

In our cohort, gestational hypertension accounted for the majority of HDP cases, followed by preeclampsia and, less frequently, eclampsia. This distribution is consistent with Mexican hospital-based data, where pregnancy-induced hypertension is predominantly classified as gestational hypertension and a smaller proportion corresponds to preeclampsia/eclampsia [39].

This retrospective cohort study identified a clear U-shaped association between age at menarche and the risk of HDP, with both early (<12 years) and late (>14 years) pubertal onset associated with a higher risk than the modal category (12–14 years). These findings complement a growing body of literature linking pubertal timing to cardiovascular regulation across the life course and provide novel evidence from a Mexican population, where this relationship had not been previously evaluated.

Our results are consistent with prior studies reporting that early menarche is associated with higher blood pressure, endothelial dysfunction, and an increased risk of hypertension later in life. Evidence from longitudinal cohorts has shown that girls who mature earlier exhibit persistently higher trajectories of adiposity, insulin resistance, and unfavorable lipid profiles throughout adolescence and adulthood [24]. Early pubertal timing has also been linked to heightened hypothalamic–pituitary–adrenal activation, sympathetic overactivity, and reduced vascular compliance, all of which have been implicated in long-term cardiovascular vulnerability [40]. Furthermore, large population-based studies, including analyses of the UK Biobank, indicate that earlier menarche is associated with higher adult blood pressure, metabolic syndrome, and cardiovascular events [37]. Mendelian randomization analyses suggest that genetically predicted earlier menarche is related to elevated systolic blood pressure and adverse cardiometabolic profiles across the life course [41]. Taken together, this body of evidence supports the interpretation that early menarche functions as a marker of earlier biological and metabolic programming, which may increase susceptibility to vascular dysregulation later in life and during pregnancy.

Conversely, and in line with evidence suggesting that deviations at either end of the pubertal spectrum may signal broader developmental vulnerability [42], the higher risk of HDP observed among women with late menarche also aligns with literature indicating that delayed pubertal onset may confer long-term cardiometabolic risk through distinct biological pathways. Late menarche has been associated with childhood undernutrition, chronic illness, and psychosocial stress—factors known to disrupt the hypothalamic–pituitary axis and delay gonadal maturation. Epidemiological studies further show that girls who mature later may exhibit persistent alterations in glucose metabolism, reduced lean mass, and adverse lipid profiles that track into adulthood [43]. Additionally, delayed puberty has been linked to lower lifetime estrogen exposure, a pattern that may impair endothelial function and vascular repair mechanisms, thereby heightening susceptibility to hypertensive complications during pregnancy [23]. Large population-based cohorts also report increased risks of ischemic heart disease and stroke among women with later menarche, reinforcing the notion that both early and late pubertal timing reflect broader developmental perturbations with enduring cardiovascular implications [44]. Taken together, these observations suggest that late reproductive maturation may constitute a distinct pathway of vascular and metabolic vulnerability, which could manifest clinically as an increased risk of hypertensive disorders during pregnancy.

In the mediation analyses, we explored whether adiposity-related mechanisms (operationalized by estimated pre-pregnancy BMI) could account for the association between age at menarche and HDP. Among women with early menarche (<12 years), the overall association with HDP was statistically significant, whereas the indirect component through pre-pregnancy BMI was small and not significant. The estimated proportion mediated was approximately 5%, suggesting that the observed association is unlikely to be explained by adult adiposity as captured by BMI. Rather than indicating a direct causal pathway, this pattern is consistent with the interpretation that early menarche may function as a marker of accelerated biological maturation and of long-term cardiometabolic sensitivity that arises earlier in life [24]. In line with this interpretation, genetic and epidemiological studies suggest that the cardiometabolic correlates of early menarche are not solely dependent on adiposity, with part of the association operating through BMI-independent pathways [41,45]. These considerations are consistent with our findings of a minimal indirect effect through pre-pregnancy BMI.

For women with late menarche (>14 years), the pattern was similar. The total effect remained significant, whereas the indirect effect through BMI was almost negligible, accounting for less than 1% of the association. This finding suggests that the observed association between late menarche and HDP is unlikely to be explained by pre-pregnancy adiposity and may reflect other underlying biological processes, such as subtle endocrine or metabolic alterations or differences in body composition not adequately captured by BM [23,43,46]. Taken together, our results indicate that the associations observed for both early and late menarche are not mediated by adult adiposity. Instead, pubertal timing at both extremes may serve as a marker of distinct developmental trajectories associated with vascular vulnerability during pregnancy, consistent with epidemiological and genetic models that conceptualize the timing of puberty as an integrated marker of complex neuroendocrine and metabolic processes [44,45].

The mediation analysis using gestational diabetes as a potential mediator indicated that only a modest proportion of the association between early menarche and HDP could be accounted for by this factor. This limited indirect effect is biologically plausible, given that gestational diabetes is associated with endothelial dysfunction, oxidative stress, and placental maladaptation processes that have been implicated in the development of HDP [47,48]. Moreover, gestational diabetes is a well-established risk factor for both gestational hypertension and preeclampsia [49,50]. The relatively small magnitude of the indirect effect suggests that the association between early menarche and HDP is only partially related to gestational diabetes and may reflect additional metabolic or developmental factors not captured by this mediator.

This interpretation is consistent with previous evidence showing that earlier pubertal timing is associated with long-term metabolic alterations, including increased adiposity, insulin resistance, and a higher risk of type 2 diabetes, factors linked to vascular vulnerability during pregnancy [24,37]. In contrast, for late menarche, the absence of an indirect effect through gestational diabetes suggests that this metabolic condition is unlikely to play a significant mediating role in the observed association with HDP. This finding supports the possibility that the relationship between late pubertal timing and HDP may involve alternative biological or developmental processes not captured by gestational diabetes in the present analysis.

### Limitations

Our study has some limitations that should be considered when interpreting the findings. First, age at menarche should be construed as a reproductive marker rather than a direct causal determinant of HDP. Menarcheal timing integrates multiple genetic, nutritional, metabolic, and psychosocial influences accumulated across childhood and adolescence, many of which could not be directly measured in this retrospective study. Consequently, the observed associations likely reflect broader developmental trajectories rather than the isolated effect of menarcheal age itself.

Although age at menarche was obtained retrospectively from clinical records, menarche represents a salient and meaningful biological milestone in women’s lives. Previous studies have shown that self-reported age at menarche is generally recalled with reasonable accuracy, even decades after its occurrence. Nevertheless, some degree of nondifferential misclassification cannot be ruled out and may have attenuated the observed associations toward the null [51].

Second, because the study relied on retrospective clinical records, some degree of exposure or outcome misclassification is possible. Importantly, any such misclassification would almost certainly be non-differential, given that diagnoses and reproductive history were documented as part of routine clinical care and without knowledge of the study hypothesis. Therefore, although measurement error cannot be completely ruled out, it is unlikely to have introduced systematic information bias.

Third, the reconstructed prepregnancy BMI represents an approximation rather than a precise measurement and reflects adult adiposity rather than body composition at the time of menarche. While this approach inevitably introduces some uncertainty, it provides a practical and reasonable estimate of baseline nutritional status in contexts where complete prenatal anthropometric data are not systematically recorded. Accordingly, mediation analyses involving prepregnancy BMI should be interpreted as exploratory and limited to the available measurements.

Fourth, despite careful adjustment for sociodemographic, obstetric, and clinical covariates, residual confounding cannot be excluded. Important early-life factors—such as childhood nutritional status, growth trajectories, physical activity during adolescence, psychosocial stress, maternal age at menarche, and genetic background—were not available in the clinical records and therefore could not be evaluated. Nevertheless, the magnitude of confounding required to explain our findings appears relatively large. For early menarche, the E-value for the adjusted point estimate was 3.03, and the lower confidence limit was 2.21; for late menarche, the corresponding E-values were 2.73 and 1.70, respectively. These estimates suggest that only moderately strong unmeasured confounding could negate the observed associations.

Finally, the observational nature of the study precludes definitive causal inference, including for the evaluated mediation pathways. Nonetheless, the large sample size, consistent patterns across multiple analytic approaches, and the use of flexible spline models strengthen the robustness and internal consistency of the findings.

## 5. Conclusions

In this retrospective cohort, age at menarche showed a consistent U-shaped association with HDP. These findings do not support a causal role of menarcheal timing itself but instead suggest that pubertal timing may serve as a stable reproductive marker reflecting earlier-life developmental processes relevant to cardiovascular adaptation during pregnancy. From a clinical perspective, age at menarche (simple to obtain and stable across the life course) may provide contextual information when assessing hypertensive risk, particularly in settings where detailed early-life data are unavailable. From a developmental perspective, the reproducible U-shaped pattern suggests that pubertal timing may reflect early-life processes relevant to vascular health. Further research incorporating hormonal, genetic, and longitudinal data is needed to clarify these pathways.

## Figures and Tables

**Table 1:** General characteristics of the study population.

Characteristics	Hypertensive Disorders of Pregnancy			*p*-Value ^a^
	n = 1344	No n = 1090	Yes n = 254	
Age (in years)				
Median (IQR)	25 (10)	24 (9)	25 (11)	0.018
Education level, *f* (%)				
Primary education	133 (9.9)	106 (9.7)	27 (10.6)	0.967
Lower secondary education	696 (51.8)	566 (51.9)	130 (51.2)	
Upper secondary education	286 (21.3)	231 (21.2)	55 (21.7)	
Tertiary education	229 (17.0)	187 (17.2)	42 (16.5)	
Marital status, *f* (%)				
No partner	38 (2.8)	27 (2.5)	11 (4.3)	0.108
With partner	1306 (97.2)	1063 (97.5)	243 (95.7)	
Monthly household income ^b^				
Median (IQR)	512 (73.6)	457.7 (75.1)	459.5 (74.8)	0.083
Pregestational BMI, *f* (%)				
BMI < 25	784 (58.3)	647 (59.4)	137 (53.9)	0.115
BMI ≥ 25	560 (41.7)	443 (40.6)	117 (46.1)	
Parity, *f* (%)				
Nulliparous	571 (42.5)	473 (43.4)	98 (38.6)	0.264
1–2	632 (47.0)	508 (46.6)	124 (44.8)	
≥3	141 (10.5)	109 (10.0)	32 (12.6)	
Previous HDP				
No	1258 (93.6)	1039 (95.3)	219 (86.2)	0.001
Yes	86 (6.4)	51 (4.7)	35 (13.8)	
Gestational diabetes				
No	1277 (95.0)	1050 (96.3)	227 (89.4)	0.001
Yes	67 (5.0)	40 (3.7)	27 (10.6)	
Alcohol consumption, *f* (%)				
No	1270 (94.5)	1030 (94.5)	240 (95.0)	0.996
Yes	74 (5.5)	60 (5.5)	14 (5.5)	
Cigarette smoking, *f* (%)				
No	1203 (89.5)	975 (89.5)	228 (89.8)	0.548
Yes	141 (10.5)	115 (10.5)	26 (10.2)	
Family history of diabetes, *f* (%)				
No	986 (73.4)	804 (73.8)	182 (71.6)	0.494
Yes	358 (26.6)	286 (26.2)	72 (28.4)	
Family history of hypertension, *f* (%)				
No	1025 (76.3)	831 (76.2)	194 (76.4)	0.962
Yes	319 (23.7)	259 (23.8)	60 (26.6)	
Age of menarche, *f* (%)				
<12 years	821 (61.1)	702 (64.4)	119 (46.9)	0.021
12–14 years	361 (26.9)	267 (24.5)	94 (37.0)	
>14 years	162 (12.0)	121 (11.1)	41 (16.1)	

Abbreviations: IQR, interquartile range; f, frequency. ^a^ Categorical variables were compared using Pearson’s chi-squared or Fisher’s exact test, and medians with the Mann–Whitney U test. ^b^ American dollars.

**Table 2:** Risk ratios for the association between age at menarche and hypertensive disorders of pregnancy.

Age at Menarche	Crude Risk (%)	RR (95% CI)	*p*-Value	RR (95% CI) ^a^	*p*-Value
12–14 years	14.4	Ref.	-	Ref.	-
<12 years	26.0	1.79 (1.41, 2.28)	0.001	1.81 (1.42, 2.30)	0.001
>14 years	25.3	1.74 (1.27, 2.38)	<0.001	1.74 (1.27, 2.37)	<0.001

Abbreviations: RR, Relative risk; CI, confidence interval; Ref., reference. ^a^ All models were adjusted for age, education, monthly household income, and family history of hypertension.

## Data Availability

The data that support the findings of this study are openly available in Mendeley Data at doi: 10.17632/wxhp3swcmy.1; https://data.mendeley.com/datasets/wxhp3swcmy/1 (accessed on 22 November 2025).

## Supplementary Materials

The following supporting information can be downloaded at: https://www.mdpi.com/article/10.3390/clinpract16020032/s1, Figure S1: Flow diagram of participant selection for the retrospective cohort study on age at menarche and hypertensive disorders of pregnancy; Figure S2. Directed acyclic graph (DAG) for the association between age at menarche and hypertensive disorders of pregnancy; Table S1. Model specification and implementation; Table S2. Sensitivity analysis of the association between age at menarche and hypertensive disorders of pregnancy using alternative categorical definitions of age at menarche (<11, 11–14, and >14 years); Table S3. Sensitivity analysis of the association between age at menarche and hypertensive disorders of pregnancy using 12–13 years as the reference category (≤11, 12–13, and ≥14 years); Table S4. Adjusted risk ratios for hypertensive disorders of pregnancy according to age at menarche, stratified by parity; Table S5. Adjusted risk ratios for hypertensive disorders of pregnancy according to age at menarche and pre-pregnancy BMI category.

## Abbreviations

The following abbreviations are used in this manuscript:

HDP	Hypertensive disorders of pregnancy
RR	Relative Risk
CI	Confidence interval
RCS	Restricted cubic splines
BMI	Body Mass Index
DAG	Directed acyclic graphs
IQR	Interquartile range
