# Supplementary material for: Age at Menarche and Risk of Hypertensive Disorders of Pregnancy: A Retrospective Cohort Study
Source: Clin Pract. 2026 Jan 29;16(2):32. doi: 10.3390/clinpract16020032 (PMC12939861; doi:10.3390/clinpract16020032)
Supplement: Supplementary file 1 [file clinpract-16-00032-s001.zip › FigureS1_Flow Chart.pdf]

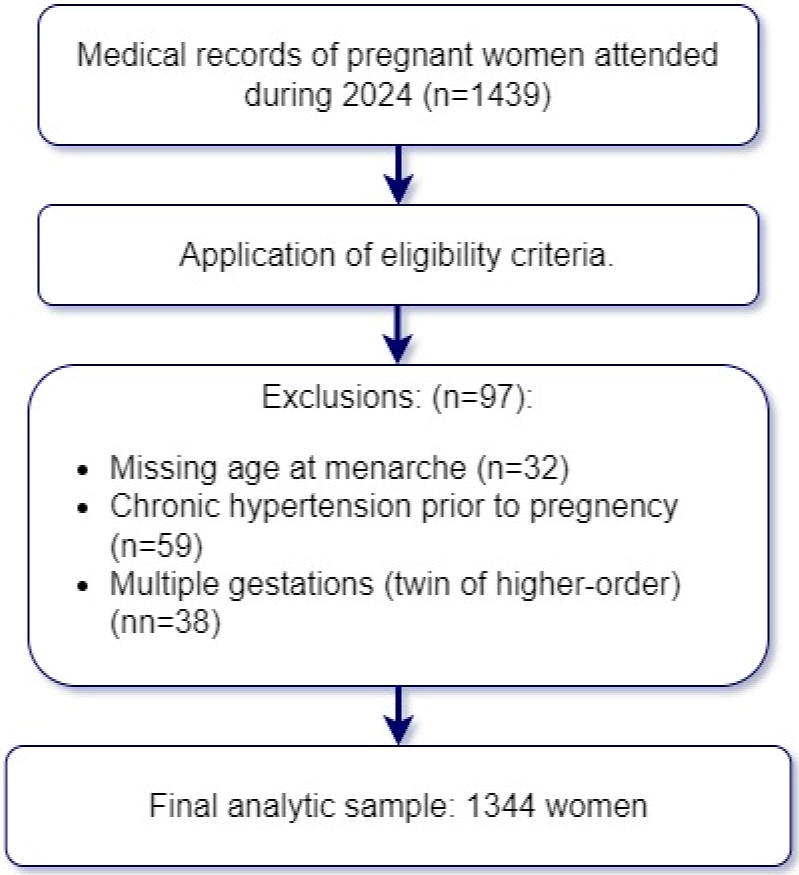

Figure S1. Flow diagram of participant selection for the retrospective cohort study on age at menarche and hypertensive disorders of pregnancy.
