# Supplementary material for: Age at Menarche and Risk of Hypertensive Disorders of Pregnancy: A Retrospective Cohort Study
Source: Clin Pract. 2026 Jan 29;16(2):32. doi: 10.3390/clinpract16020032 (PMC12939861; doi:10.3390/clinpract16020032)
Supplement: Supplementary file 1 [file clinpract-16-00032-s001.zip › Table S1.pdf]

Supplementary Table S1. Model specification and implementation.

Model Formulas

- **Main adjusted Poisson regression model (categorical exposure)**

  log{E(Yi)} = β0 + β1 Menarchei + β2 MaternalAgei + β3 Educationi + β4 Incomei + β5 FamilyHistoryHTNi

  where Yi indicates the presence of hypertensive disorders of pregnancy (HDP), and Menarche is modeled as a categorical variable (<12, 12–14 [reference], >14 years).

- **Restricted cubic spline model (continuous exposure)**

  log{E(Yi)} = β0 + f(Menarchei) + Σ βk Xik

  where f(·) represents a restricted cubic spline function for age at menarche with four knots located at 10, 11, 13, and 15 years (5th, 35th, 65th, and 95th percentiles, respectively), and Xik denotes the adjustment covariates.

Stata code for model implementation [a]

- **Main analysis: modified Poisson regression with robust variance**

```
poisson hipertension i.menarca2 matage i.womeduc income antecedentehta, vce(robust)
```

- **Restricted cubic splines for age at menarche**

```
* Knot locations (years): 10, 11, 13, 15
mkspline mrcs = menarca, cubic knots(10 11 13 15)
poisson hipertension mrcs* matage i.womeduc income antecedentehta, vce(robust)
*Test for non-linearity
test mrcs2 mrcs3
```

[a] **Variable definitions used in the statistical code**: hipertension, hypertensive disorders of pregnancy (binary outcome); menarca, age at menarche (years); menarca2, age at menarche categorized as <12, 12–14 (reference), and >14 years; matage, maternal age at delivery (years); womeduc, maternal educational level (categorical); income, monthly household income; antecedentehta, family history of hypertension (yes/no); imcpre_cat, pre-pregnancy body mass index categorized as <25 vs. ≥25 kg/m^2; paridad2, parity categorized as nulliparous vs. multiparous
