# Supplementary material for: Age at Menarche and Risk of Hypertensive Disorders of Pregnancy: A Retrospective Cohort Study
Source: Clin Pract. 2026 Jan 29;16(2):32. doi: 10.3390/clinpract16020032 (PMC12939861; doi:10.3390/clinpract16020032)
Supplement: Supplementary file 1 [file clinpract-16-00032-s001.zip › Table S2.pdf]

**Table S2. Sensitivity analysis of the association between age at menarche and hypertensive disorders of pregnancy using alternative categorical definitions of age at menarche (<11, 11–14, and >14 years).**

| Age at menarche | hypertensive disorders of pregnancy | | | |
|---|---|---|---|---|
| | RR (CI 95%) | P-value | RR (CI 95%) [a] | P-value |
| 11-14 years | Ref. | - | Ref. | - |
| < 11 years | 1.47 (1.15, 1.88) | 0.002 | 1.49 (1.16, 1.90) | 0.001 |
| > 14 years | 1.51 (1.09, 2.07) | 0.010 | 1.50 (1.09, 2.06) | 0.012 |

Abbreviations: RR, Risk ratio; CI, confidence interval; Ref, reference.

[a] All models were adjusted for age, education, monthly household income, and family history of hypertension
