# Supplementary material for: Age at Menarche and Risk of Hypertensive Disorders of Pregnancy: A Retrospective Cohort Study
Source: Clin Pract. 2026 Jan 29;16(2):32. doi: 10.3390/clinpract16020032 (PMC12939861; doi:10.3390/clinpract16020032)
Supplement: Supplementary file 1 [file clinpract-16-00032-s001.zip › Table S3.pdf]

Table S3. Sensitivity analysis of the association between age at menarche and hypertensive disorders of pregnancy using 12‑13 years as the reference category (≤11, 12‑13, and ≥14 years).

Age at menarche	Hypertensive disorders of pregnancy			
	**RR (CI 95%)**	**P-value**	**RR (CI 95%)** [a]	**P-value**
12-13 years	Ref.	-	Ref.	-
≤ 11 years	1.79 (1.38, 2.30)	0.012	1.78 (1.38, 2.30)	0.001
≥ 14 years	1.53 (1.15, 2.03)	0.925	1.49 (1.12, 1.99)	0.006

Abbreviations: Abbreviations: RR, Risk ratio; CI, confidence interval; Ref, reference

[a] All models were adjusted for age, education, monthly household income, and family history of hypertension
