# Supplementary material for: Age at Menarche and Risk of Hypertensive Disorders of Pregnancy: A Retrospective Cohort Study
Source: Clin Pract. 2026 Jan 29;16(2):32. doi: 10.3390/clinpract16020032 (PMC12939861; doi:10.3390/clinpract16020032)
Supplement: Supplementary file 1 [file clinpract-16-00032-s001.zip › Table S4.pdf]

Table S4. Adjusted risk ratios for hypertensive disorders of pregnancy according to age at menarche, stratified by parity

Parity	Age at menarche	Adjusted RR (95% CI)	p-value
Nulliparous	<12 vs 12-14	2.03 (1.39, 2.98)	<0.001
Nulliparous	>14 vs 12-14	1.68 (0.96, 2.95)	0.068
Multiparous	<12 vs 12-14	1.68 (1.23, 2.29)	0.001
Multiparous	>14 vs 12-14	1.76 (1.21, 2.57)	0.003

Abbreviations: RR, risk ratio; CI, confidence interval

Footnote: Footnote: Parity was dichotomized as nulliparous vs multiparous to preserve statistical power. Adjusted risk ratios were estimated using Poisson regression models with robust variance. All models were adjusted for maternal age, educational level, monthly household income, and family history of hypertension. p-values correspond to Wald tests for each stratum-specific contrast.

P-values correspond to Wald tests for each stratum-specific contrast.
