# Supplementary material for: Age at Menarche and Risk of Hypertensive Disorders of Pregnancy: A Retrospective Cohort Study
Source: Clin Pract. 2026 Jan 29;16(2):32. doi: 10.3390/clinpract16020032 (PMC12939861; doi:10.3390/clinpract16020032)
Supplement: Supplementary file 1 [file clinpract-16-00032-s001.zip › Table S5.pdf]

Table S5. Adjusted risk ratios for hypertensive disorders of pregnancy according to age at menarche and pre-pregnancy BMI category.

BMI stratum	Age at menarche	Adjusted RR (95% IC)	p-value
<25 kg/m²	<12 vs 12-14	1.90 (1.36, 2.66)	<0.001
<25 kg/m²	>14 vs 12-14	1.96 (1.30, 2.95)	0.001
≥25 kg/m²	<12 vs 12-14	1.63 (1.15, 2.31)	0.006
≥25 kg/m²	>14 vs 12-14	1.47 (0.91, 2.31)	0.115

Abbreviations: RR, risk ratio; CI, confidence interval

Footnote: Adjusted risk ratios were estimated using Poisson regression models with robust variance.

All models were adjusted for maternal age, educational level, monthly household income, and family history of hypertension. p-values correspond to Wald tests for each stratum-specific contrast.

The global test for multiplicative interaction between age at menarche and pre-pregnancy BMI was not statistically significant (p = 0.63)
